# Exploring potential applications of measles and rubella microarray patches (MR-MAPs): use case identification

**DOI:** 10.3389/fpubh.2023.1165110

**Published:** 2023-06-12

**Authors:** Stefano Malvolti, Melissa Ko, Marion Menozzi-Arnaud, Carsten Mantel, Courtney Jarrahian, Jean-Pierre Amorij, Birgitte Giersing, Mateusz Hasso-Agopsowicz

**Affiliations:** ^1^MMGH Consulting GmbH, Zurich, Switzerland; ^2^Gavi, The Vaccine Alliance, Geneva, Switzerland; ^3^PATH, Seattle, WA, United States; ^4^Supply Division, Vaccine Centre, UNICEF, Copenhagen, Denmark; ^5^Immunisation, Vaccines and Biologicals, World Health Organization, Geneva, Switzerland

**Keywords:** measles, rubella, microarray patches, use cases, forecast

## Abstract

**Introduction:**

Innovative vaccine products will be critical in helping to address the existing implementation barriers that have prevented the achievement of the measles and rubella (MR) vaccine coverage targets. Overcoming those barriers will be necessary to achieve the “Immunization Agenda 2030” goals. Microarray patches (MAPs), an innovative needle-free delivery device currently in clinical development, can be a potential game changer in this respect and contribute to the equitable delivery of vaccines in low- and middle-income countries and pandemic preparedness and response. Developing in-depth knowledge of the most desired and impactful uses of MRMAPs can prove critical to identifying the critical attributes of the target product profile, informing policy and adoption decisions, and helping to evaluate the potential public health and economic value of this technology. The first step in this process is the definition of the potential use cases for MR-MAPs, i.e., where and how this product is most likely to be used within the immunization programme.

**Methods:**

By applying a design-based user-centric approach, we implemented a three-step process, including a desk review, a survey, and interviews, to define the most relevant use cases for MR MAPS.

**Results:**

Six use cases have been identified as relevant across all different countries and immunization programme designs and validated by experts.

**Discussion:**

The identified use cases have already informed the demand estimate for MR-MAPs and provided the foundation for developing an initial full vaccine value assessment. We believe that, in the future, they will be highly valuable in ensuring that the roll-out of this promising innovation is designed in a way that maximizes the impact, particularly in populations and countries that are most in need.

## 1. Introduction

Measles is an acute respiratory disease caused by the measles virus that results in severe morbidity and mortality, particularly in infants living in settings with poorly functioning health systems. Its high level of transmissibility makes the control of this disease especially challenging (1). Rubella is caused by the rubella virus and is transmitted via the respiratory route. Although clinically mild, a primary infection just before conception and during the first 8–10 weeks of gestation may result in a miscarriage or a child born with congenital rubella syndrome, which is the most common infectious cause of congenital disabilities and may result in multiple fetal defects in up to 90% of cases, or severe developmental disabilities (2). Owing to immunization, the annual number of measles deaths has dropped by 94% between 2000 and 2020 (3), and a similar impact on rubella incidence has been observed in countries that have introduced the rubella vaccine (4).

The COVID-19 pandemic caused a sudden reversal in these positive trends. It is estimated that approximately 9 million children worldwide missed the first dose of the measles-rubella vaccine in 2020 (5). As a result, only in the first 2 months of 2022 did the number of reported measles cases increase by 79% compared to the same period in 2021 (6, 7).

To increase measles and rubella (MR) vaccine coverage and prevent further backsliding, the “Immunization Agenda 2030” (8) and the “Measles and Rubella strategic framework: 2021–2030” identified research and development of novel vaccine product innovations as critical to achieving high measles and rubella population immunity and progress toward the elimination of measles and rubella disease (9, 10). Similarly, the Vaccine Innovation Priority Strategy (VIPS), an initiative co-led by Gavi, the Vaccine Alliance (Gavi), the World Health Organization (WHO), and the United Nations Children's Fund (UNICEF), together with the Bill and Melinda Gates Foundation (BMGF) and PATH, prioritized microarray patches (MAPs), a novel delivery device in development that may be potential game changers for the equitable delivery of vaccines in low- and middle-income countries (LICs and MICs) and contribute to pandemic preparedness and response (11).

MAPs are needle-free delivery devices consisting of up to hundreds or thousands of tiny projections that deliver dry vaccines just below the skin surface, with some MAPs applied manually and others requiring an applicator for delivery. The vaccine is delivered within seconds to a few minutes of application, and the patch can then be discarded, most likely as biohazardous waste. MAP presentations and their characteristics have potential advantages over the current vaccine presentation of needles and syringes. MR-MAPs are anticipated to be a single dose, remove the need for reconstitution, and have enhanced heat stability. These characteristics can help address key technical MR vaccine delivery challenges, such as reducing vaccine wastage and potential programmatic errors, improving safety, removing sharps waste, reducing cold chain requirements, and increasing acceptance. As such, MR-MAPs are perceived to significantly ease the delivery of MR vaccines, improve the user experience, and enhance equitable coverage of MR vaccines. As such, in 2016, WHO's Strategic Advisory Group of Experts on Immunization (SAGE) recommended identifying the most expeditious pathway to development for MR-MAPs (12).

Despite the product advantages and anticipated impact on MR immunization programmes, MR-MAPs have only recently entered clinical development, with the first Phase IIb data anticipated in early 2023. With committed and at-risk investment in manufacturing and late-stage development, MR-MAPs could become available to countries before 2030 (13). To accelerate the development of the innovation, WHO, UNICEF, and a group of MR experts published an MR-MAP Target Product Profile (TPP) (14), containing a list of attributes that are needed for MR-MAPs to make an impact in LICs and MICs. In addition, WHO and its partners have estimated the demand for MR-MAPs between 2030 and 2040 (15), while UNICEF evaluated the value proposition of this innovative presentation (the work will be made public in early 2023). A critical component underpinning all these analyses is an evaluation of the potential use cases of MR-MAPs, i.e., where and how the innovation is envisaged to be used within the immunization programme. Knowledge of the most desired and impactful uses of MR-MAPs will help identify the critical attributes of the TPP, inform the direction of the demand forecast, and help evaluate the potential public health and economic value of this technology.

This article describes the research conducted to evaluate the potential use cases of MR-MAPs as a basis for the ongoing workstreams, with the goal of expediting the development of these innovations.

## 2. Methodology to define MR-MAP vaccine use cases

### 2.1. Definition of use case

A use case, a concept originating from software development (16), describes “*a specific situation where a product or a service is or can be used to achieve a stated goal*.” For vaccines, it has been recognized that research to understand the context and perspectives with regards to the use of a product (use cases) is needed to focus attention on all aspects that can facilitate or derail product utilization and to use this information to guide product development and ensure future optimal and rapid deployment. The methodology described in this article has been validated by the WHO's MR-MAP Working Group and WHO's Immunization and Vaccines-related Implementation Research Advisory Committee (IVIR-AC) in September 2020 (17).

### 2.2. Development of use cases

The process of defining the use cases involved three steps.

First, a **landscape analysis** of MR-MAP's technical and programme feasibility and acceptability was performed to identify the most relevant dimensions for defining the use cases. This included a desk review covering a variety of relevant areas: measles and rubella epidemiology across countries, the MAP product pipeline, the challenges facing vaccine and MAP product development, the potential programmatic impact of MAP vaccines, the challenges with the deployment of existing MR vaccines, and end-user acceptability studies. The emerging findings were documented and discussed with a group of experts assembled to guide the process, and potential initial use cases were defined.

In the second step, in March 2020, a **survey** was administered to 111 immunization stakeholders, representatives of the various groups currently and potentially interested in MR-MAP, via a personalized link through the online survey platform Qualtrics™, complemented by the posting of an anonymous link on the TechNet website to solicit other responses (methodology and key statistics on the targeted stakeholders can be found in Annex 2). The target audience included representatives from the pharmaceutical industry, MAP developers, international organizations, non-governmental organizations, representatives of ministries of health, donors, and researchers. Respondents were requested to answer questions related to a country's ability to achieve its MR control and elimination goals by (i) identifying key MR vaccine delivery challenges and influential factors, (ii) evaluating the importance of the contribution of each preliminary MR-MAP use case toward the achievement of the measles and rubella control and elimination goals; and (iii) identifying where MR-MAPs could be used and have the most contribution. All questions can be found in Annex 3. The answers, based on a 5-point scale (ranging from 0 = “not at all important” to 5 = “extremely important”), were analyzed by calculating the mean score per answer and the proportion of positive responses, as well as by individual evaluation of qualitative statements.

In the third step, immunization stakeholder **interviews** were conducted between June and July 2020 to better assess the insights emerging from the survey conducted in step 2 and to further evaluate the use of MR-MAPs in country contexts and their benefits (the methodology and responders can be found in Annex 4).

A set of countries was selected as the target for interviews based on the following factors:

- The 10 most populous countries.
- The 10 countries with the most unimmunised children, using WHO/UNICEF Estimates of National Immunization Coverage (WUENIC) for 2019 for the first dose of measles-containing vaccines (MCV1) (18).
- Countries that were previously selected for the VIPS country consultations (19).
- Countries that represent the high-priority countries for Gavi and the Measles and Rubella Initiative.
- A selection of countries that are classified as middle- or high-income countries per the World Bank (2019) and
- A selection of countries that were experiencing protracted crises.

Based on the criteria above, representatives from 49 countries, including immunization managers and WHO immunization officials, were invited to participate in an interview. The respondents answered a series of predefined questions related to (i) their current technical MR vaccine delivery challenges; (ii) whether the proposed use cases would be appropriate for the country and why; (iii) any additional use cases; and (iv) how MR-MAPs would help resolve the previously identified technical vaccine delivery challenges and contribute to the achievement of their MR goals. Questions and an overview of the preliminary use cases were sent in advance. (The interview guide can be found in Annex 5).

All interview transcripts were reviewed and discussed to identify the key results and emerging themes. TextiQ^TM^ from Qualtrics^TM^ was used to group qualitative feedback into topics and perform sentiment analysis (e.g., assigning a positive, neutral, mixed, or negative sentiment to the qualitative feedback).

The results of the landscape analysis, survey, and interviews were analyzed to revise the preliminary use cases and propose the final list of MR-MAP use cases.

## 3. Results

### 3.1. Results of the landscape analysis

The first output of the landscape analysis was the identification of different dimensions that could impact how MR-MAP vaccines would be used and ultimately define the MR-MAP use cases:

- Disease burden: Measles and rubella endemicity and co-endemicity define the public health need and may command a different attitude and sense of urgency for policymakers and decision-makers toward this innovative presentation.
- Country characteristics: Income levels will likely determine a different price sensitivity for a novel vaccine presentation that may be priced higher than needle and syringe, influencing the likelihood of adoption; the strength of the health systems may result in different delivery settings that are more or less suitable for the use of MR-MAPs vaccines (e.g., the potential to involve community health workers).
- Delivery setting: role and importance of settings with complete health services (such as hospitals or health centers) to inform the appropriate vaccine delivery strategies (e.g., routine or campaign immunization).
- Service providers: the type of service providers involved in the immunization activities–health workers (HWs), community health workers (CHWs), teachers, community leaders, caregivers, or through self-administration. Their level of training and health knowledge can influence the acceptability and effectiveness of the MR-MAPs delivery.
- Targeted population: the target age groups and their co-morbidities can all trigger changes in the way a vaccine can be used. In the case of MR vaccines, the focus on infants and young adults for campaigns reduced the “variety” of use cases that could be relevant (e.g., the viability of self-administration or the ability to leverage certain professional or educational settings).
- Vaccine product characteristics: Different schedules, routes of administration, and vaccine presentations (e.g., a lyophilised product vs. a liquid one), as well as efficacy and duration of protection, all have a significant impact on the delivery strategies and the end-user acceptability and uptake of the vaccine. These product characteristics become difficult to modify as the vaccine-MAP progresses in clinical development.

The aspects above were all discussed and agreed upon with the expert group supporting the project with the goal of selecting those dimensions that will most likely influence the use of MR-MAPs vaccines. The delivery setting, particularly with reference to the availability of the cold chain and the service provider (the vaccinator), with a specific focus on the skill level required for vaccine administration, was selected as the most important dimension and subsequently used to define the MR-MAP use cases.

**Figure 1:**
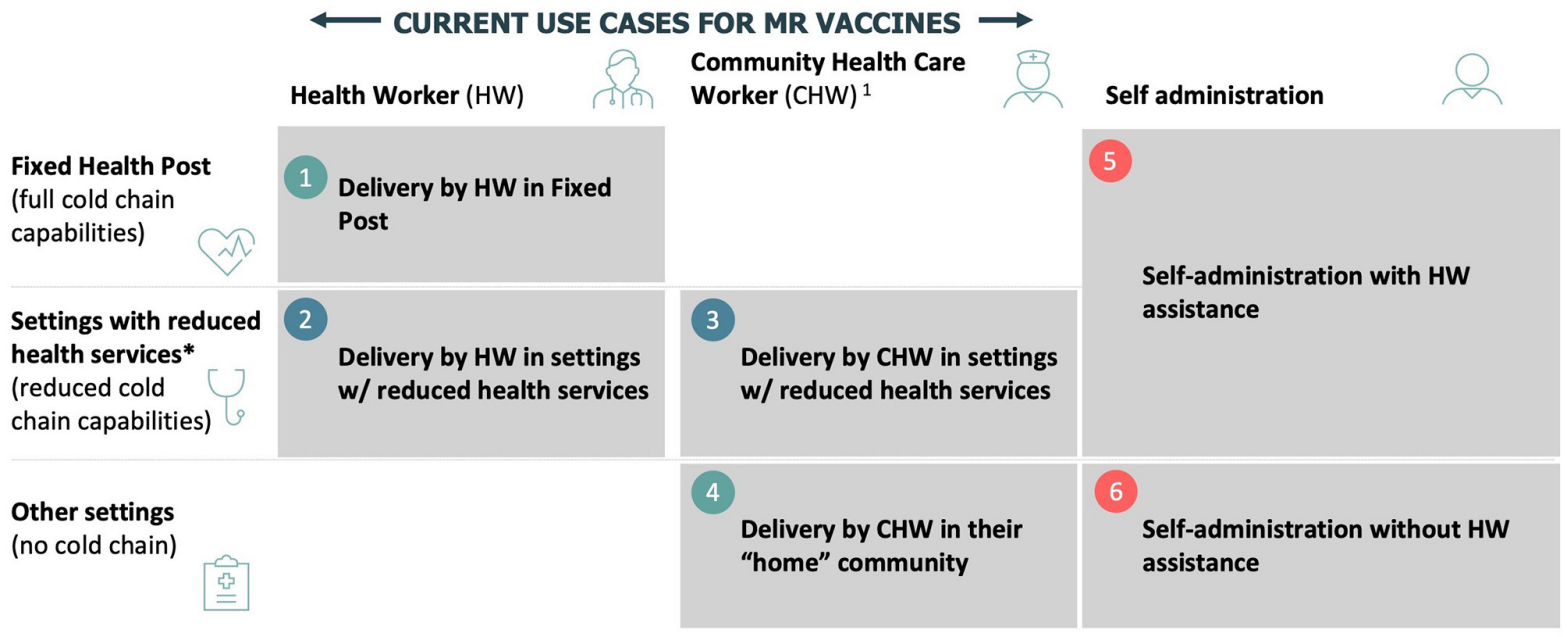
Preliminary use cases for MR-MAPs. ^*^Includes school delivery, mobile outreach, and settings with some health services. HW, health workers; CHW, community health workers.

Based on these initial findings, six draft use cases were developed and subsequently tested and validated via an online survey and interviews (Figure 1):

- Delivery in a fixed post by trained HWs or CHWs
- Delivery by trained HWs in locations with limited or no cold chain capabilities
- Delivery by CHWs in locations with limited cold chain capabilities
- Delivery by CHWs in their home community with no cold chain capabilities
- Self-administration with HWs or CHWs assistance
- Self-administration (a possibility with community members' assistance).

### 3.2. Results of the online survey

**Figure 2:**
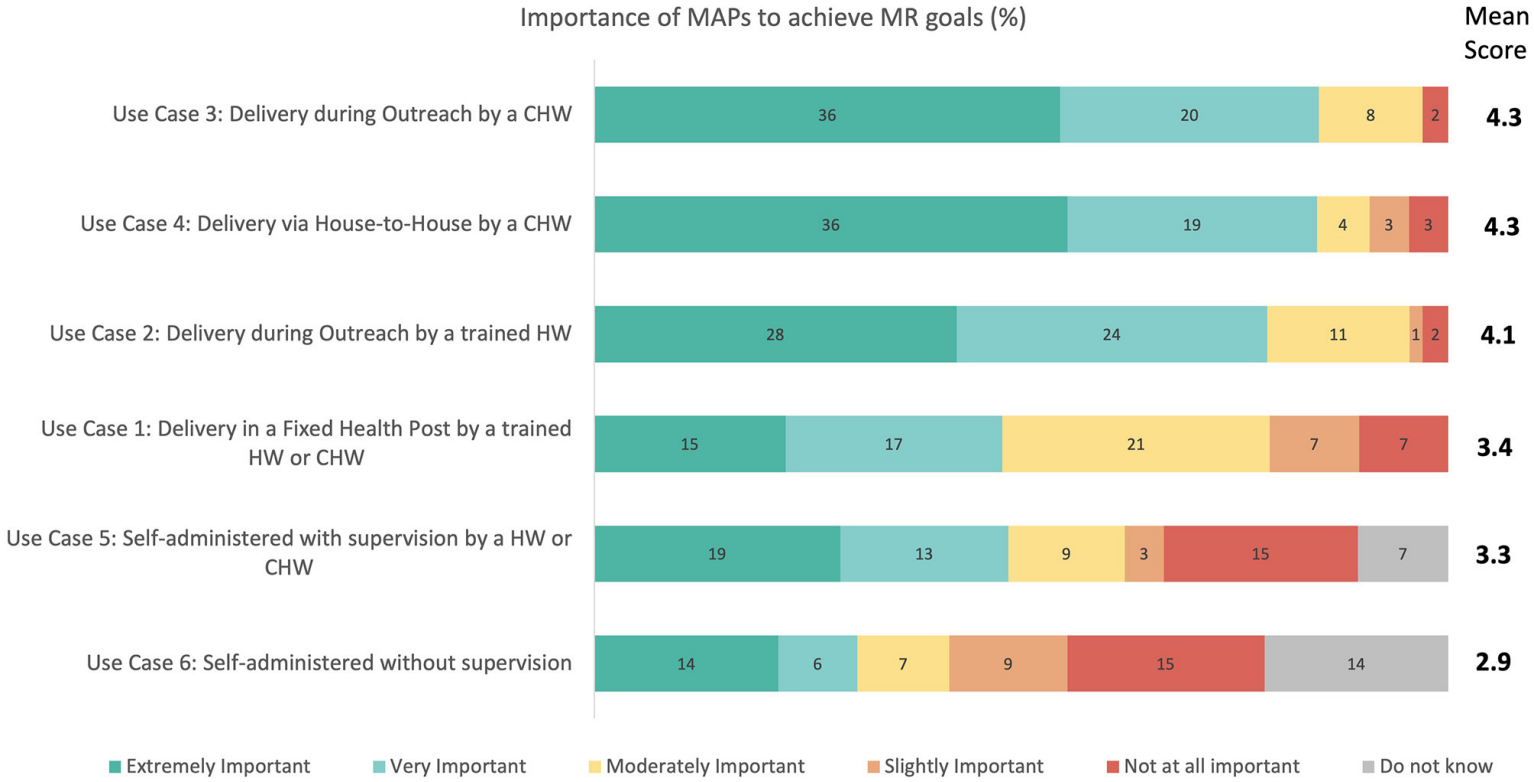
Immunization stakeholder analysis on the importance of MAPs to achieve a country's measles and rubella control and elimination goals by preliminary use cases. Number of responders = 65. MR, measles and rubella; CHW, community health workers; HW, health workers; MAPs, microarray patches.

Seventy individuals representing a variety of stakeholder groups participated in the survey and rated the importance of the preliminary MR-MAP use cases (see Annex 2 for a full breakdown of the participants). Delivery by CHWs in locations with limited cold chain capability (Use Case 3), delivery by CHWs in in-home communities with no cold chain (Use Case 4), and delivery by HWs in locations with limited or no cold chain capabilities (Use Case 2) were indicated as the most important use cases for MR-MAP. The importance of delivery by HWs in fixed health posts (Use Case 1) and self-administration with HWs assistance (Use Case 5) was rated as moderate, while self-administration with no HWs assistance (UC6) received the lowest rating. 27 and 47% of respondents stated that Use Cases 5 and 6 were only slightly or not at all important (Figure 2).

The stratified analysis by responder type showed a general agreement amongst the different perspectives for Use Cases 2, 3, and 4. However, opinions differed, particularly for the use cases utilizing self-administration (Use Cases 5 and 6) and delivery in a fixed health post (Use Case 1). Respondents with an industry background rate these use cases as more important than those representing global, regional, or national public health organizations (Annex 1, Supplementary Figure 1).

**Figure 3:**
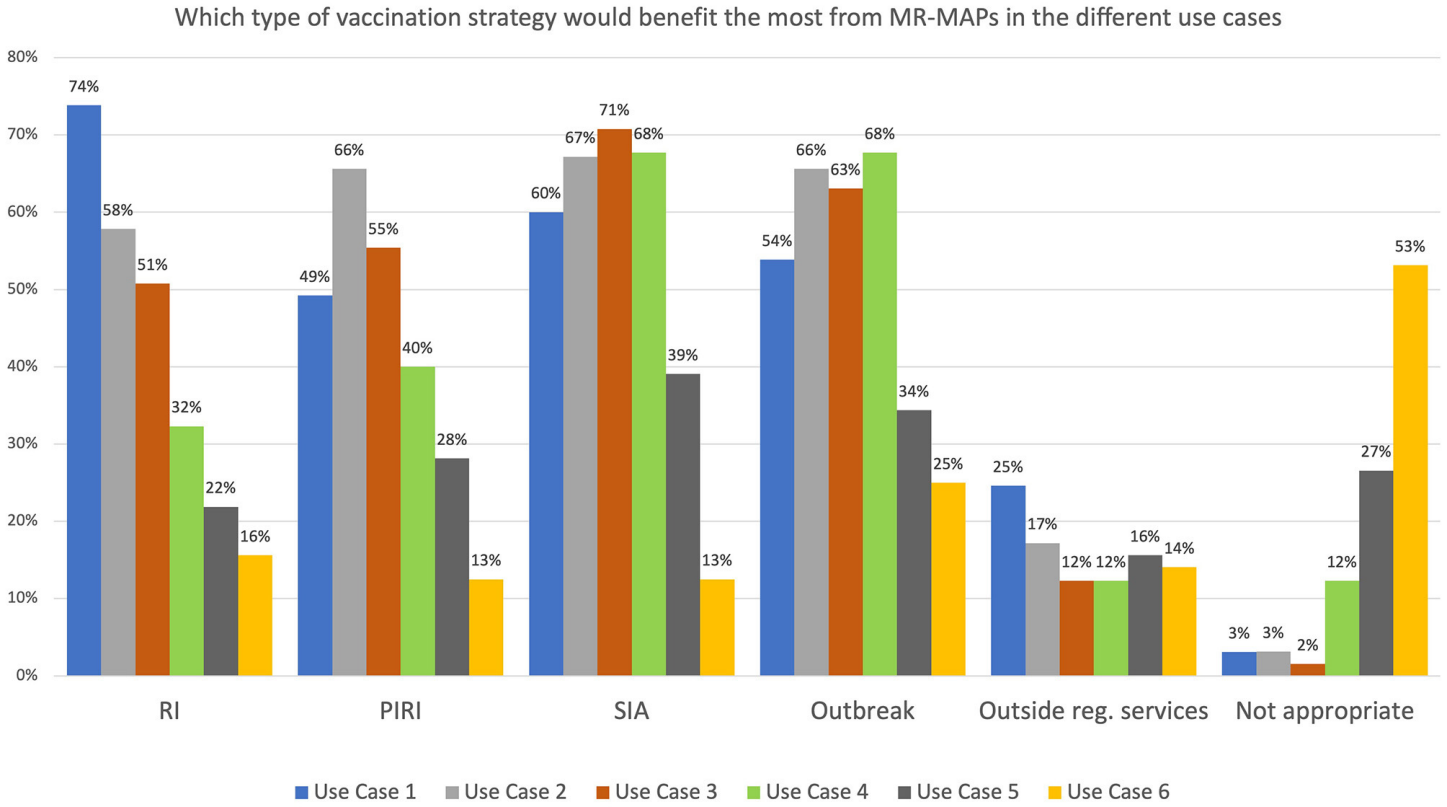
Immunization stakeholder analysis on the importance of MR-MAPs to vaccination programmes for the preliminary use cases. Number of responders = 65. HW, health worker; CHW, community health worker; w/o, without; RI, Routine Immunization; PIRI, Periodic Intensification of Routine Immunization; SIA, Supplementary Immunization Activities.

Survey participants were asked which vaccination programmes would benefit the most from MR-MAPs. More than 50% felt that Use Cases 1, 2, and 3 would be relevant for routine immunization, periodic intensification of routine immunization (PIRI), supplementary immunization activities (SIAs), and outbreak response. Most respondents felt that Use Case 4 was only relevant during SIAs and outbreak activities. For both Use Cases 5 and 6, the percentage of respondents who believed that the different vaccination strategies would benefit from MR-MAPs was below 40%, with 27 and 53% of them indicating that those use cases would not be appropriate for the setup of the immunization programme in their country, respectively. However, the respondents felt that Use Case 5 has more potential than Use Case 6 to benefit the vaccination programmes, particularly during SIAs and outbreak response vaccination (Figure 3).

**Figure 4:**
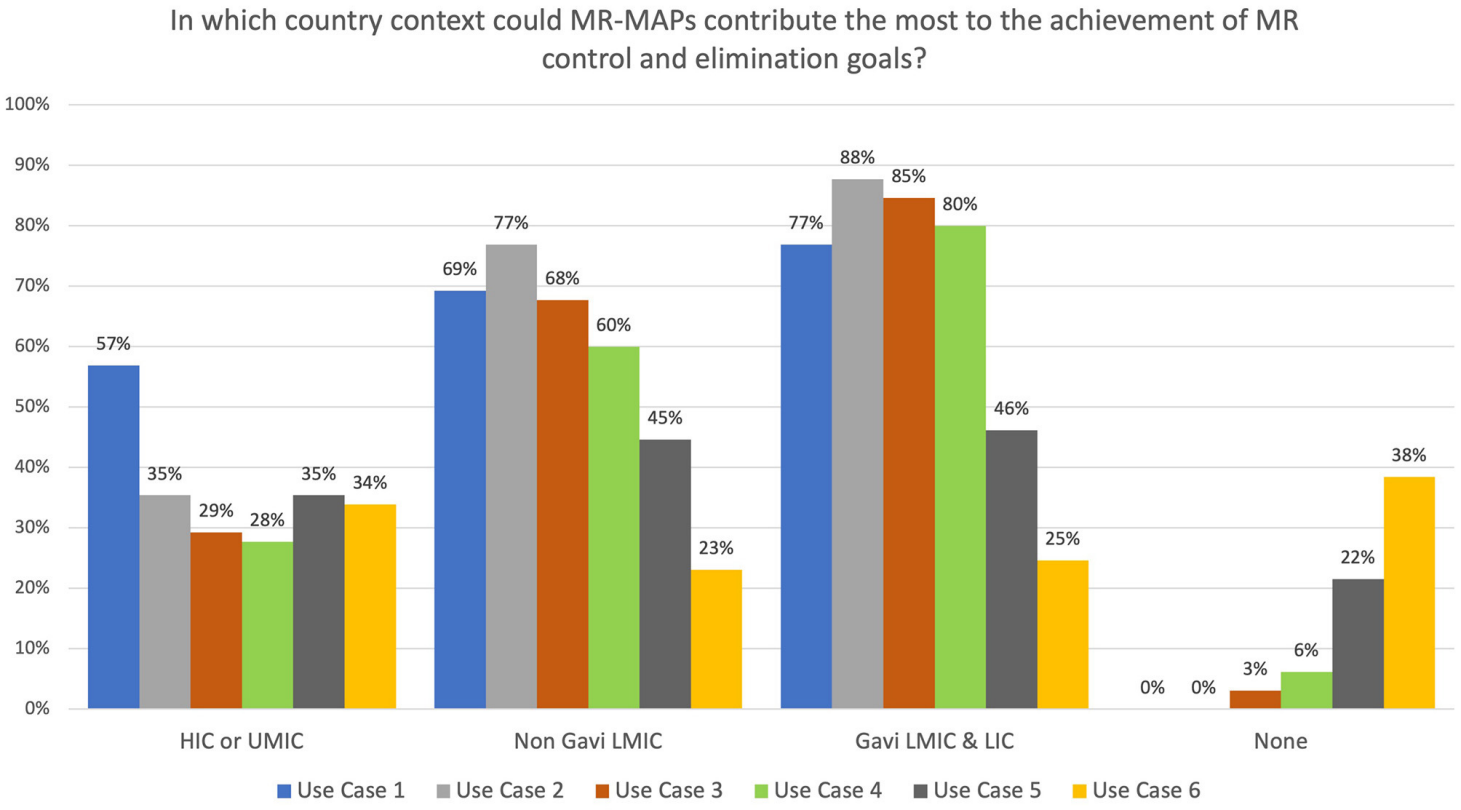
Immunization stakeholder analysis on the importance of MR-MAPs to countries for the preliminary use cases. Number of responders = 65. HICs, high-income countries; UMICs, upper middle-income countries; LMICs, lower middle-income countries; LICs, low-income countries; HW, health worker; CHW, community health worker; w/o, without.

Survey participants were asked to select the country income groups where MR-MAPs would contribute most to MR control and elimination goals. While most respondents indicated that all countries could use Case 1, opinions differed for the other use cases. Between 28 and 35% of the respondents felt that high-income and upper-middle-income countries could use MR-MAPs for all other use cases (Figure 4). With reference to LMICs, irrespective of their Gavi support status, a high percentage (between 60 and 88%) of respondents saw Use Cases 2, 3, and 4 as relevant for MR-MAPs. Approximately 45% and 25% of the respondents felt Use Cases 5 and 6 could be relevant for LMICs, irrespective of the type of Gavi support. Those responses suggested a high acceptability of Use Cases 1, 2, 3, and 4, with only a limited number of respondents indicating that those use cases would not be relevant. Conversely, Use Cases 5 and 6 were perceived as problematic by a large percentage of respondents: 22 and 38% of them felt those use cases were appropriate for delivering MR-MAPs vaccines (Figure 4).

Finally, survey respondents provided additional views related to MR-MAPs use cases, including the potential use in schools or universities, in emergency rooms, at mass gatherings, in outbreak or humanitarian settings, for reducing missed opportunities for vaccination, as part of multi-age campaigns, for use by parents on their children, and for use by travelers. As these suggestions were already covered within the preliminary use cases, such feedback was used to clarify and refine the existing definitions.

### 3.3. Results of the interviews

Twenty-nine interviews were conducted to further understand the use of MR-MAPs in countries (Annex 1, Supplementary Figure 2). Most interviewed individuals agreed that Use Cases 3 (82%) and 4 (70%) could be relevant in their countries as they saw the benefits of this presentation in allowing an expansion of the workforce, including CHWs, to deliver vaccines. A few respondents highlighted that, similarly to oral polio vaccines (OPV), MR-MAPs could be delivered by volunteers. Many also saw the benefits of improving access to hard-to-reach or security-compromised areas and the chronically un- and under-immunized. Some interviewees indicated that Use Cases 3 and 4 would not be acceptable because current policies limit the delivery of vaccines to trained health workers, often because of past MR-related serious adverse events following immunization (AEFI).

Similarly, a few respondents indicated the need to roll out MR-MAPs in fixed health posts with trained HWs to increase acceptance and trust prior to providing MR-MAPs with the help of CHWs. Respondents also indicated that Use Case 4 was less likely than Use Case 3 unless appropriate supervision existed. Finally, some respondents felt that Use Cases 1 and 2 would not be relevant for their countries as they utilized measles, mumps, rubella (MMR) and MMR plus varicella (MMRV) vaccines in their routine schedules or had strong delivery systems in place, not warranting changes that could have negative implications for the delivery of other vaccines.

Although most respondents were initially cautious toward Use Cases 5 and 6, the potential contribution of those use cases became clearer upon further discussion. Most respondents cited two key barriers to self-administration: the inability to record, report, and track vaccinations and the inability to monitor and report AEFIs. Regardless, 50% of the respondents had a positive view on the viability of Use Case 5, citing that older age groups could self-administer MR-MAPs under HWs or CHWs supervision, or MR-MAPs could be used for self-administration in hard-to-reach, security-compromised, or remote areas. Three country respondents preferred Use Case 5 over all other use cases. In contrast, others cited global pandemics such as COVID-19 as providing potential opportunities for Use Case 5 (e.g., provision of the MR-MAP vaccine to the caregiver for administration under the supervision of an HW, hence eliminating the need for proximity between the vaccinator and the vaccinee/caregiver).

The viability of Use Case 6 remained the most problematic, with some countries indicating that it might be possible only following appropriate and extensive advocacy and communications and in circumstances where communities feel responsible for and proud of utilizing innovative technology. Respondents indicated that MR-MAPs could become a powerful tool against hesitancy as parents could administer the vaccine themselves, thus becoming directly involved in the vaccination process and feeling more empowered. At the same time, other respondents indicated that Use Case 6 would not be a viable solution because it would prevent combining MR vaccine administration with other vaccinations and other infant and child interventions normally delivered in the same visit and requiring HW intervention.

### 3.4. The final definition of MR-MAP use cases

**Figure 5:**
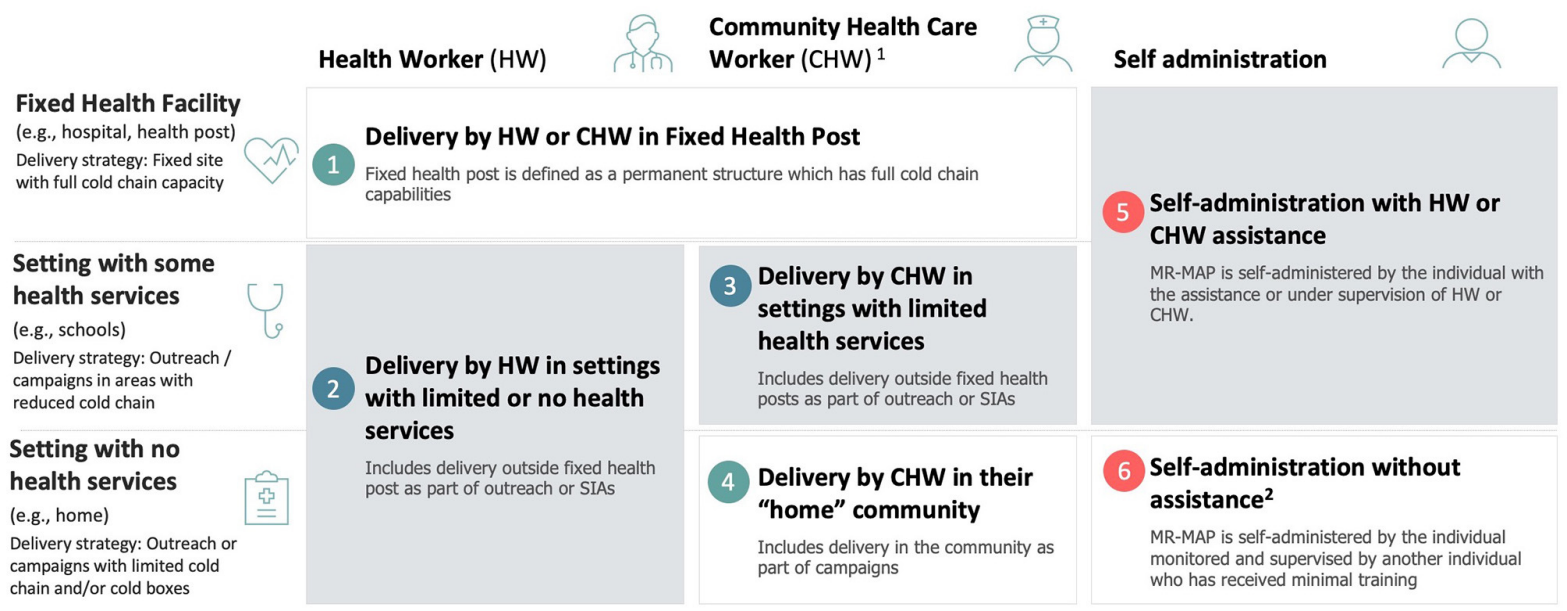
Revised and finalized definition of MR-MAPS use cases.1: CHWs provide health education, referral and follow-up, case management and basic preventive health care and home visiting services to specific communities. They provide support and assistance to individuals and families in navigating the health and social services system. Occupations included in this category normally require formal or informal training and supervision recognized by the health and social services authorities; 2: This may include community member assistance (e.g., teachers, elders, etc.) who have not been trained in MAPs but can monitor and document the administration. HW, health worker; CHW, community health worker; SIAs, supplementary immunization activities; MR-MAP, measles and rubella microarray patch.

The results of the surveys, interviews, and archetype analysis served as a base for refining the use cases and formalizing a final set of definitions (Figure 5):

- Use Case 1: Delivery by a health worker or community health worker in a fixed health post. MR-MAP is delivered by trained HWs in a permanent structure that has full cold chain capabilities. *Examples: public and private hospitals and health facilities at all service delivery levels*.
- Use Case 2: Delivery by a health worker in settings with limited or no health services. MR-MAP is delivered by trained HWs, in areas that do not have access to a fixed health post and have reduced or no cold chain capacities as part of outreach or campaigns. *Examples: outreach by HWs to remote or hard-to-reach areas in the catchment area of fixed health posts as part of school vaccination, reactive or pre-emptive vaccination, outbreak response immunization, and so on*.
- Use Case 3: Delivery by a community health worker in settings with limited health services. MR-MAP is delivered by CHWs in remote areas far from fixed health posts and with reduced or no cold chain capacity. *Examples: outreach by CHWs to remote or hard-to-reach areas in the catchment area of fixed health posts, including school vaccination, reactive or pre-emptive vaccination, outbreak response immunization, and so on*.
- Use Case 4: Delivery by a community health worker in their “home” community. MR-MAPs are delivered by CHW in the area where they live–in the community–with highly limited or no cold chain capacity. *Examples: remote areas, security-compromised areas, or areas that are inaccessible during specific times due to annual weather patterns (e.g., flooding)*.
- Use Case 5: Self-administration with a health worker or community health workers' assistance. MR-MAP is self-administered by the individual or administered by a caregiver in a health post or in a setting with some health services with the assistance or supervision of an HW or CHW, who can monitor for AEFI and record and report those who have received the vaccination. *Examples: school vaccination, outbreak vaccination, vaccination during COVID-19, social distancing situations, including parent-to-child, and so on*.
- Use Case 6: Self-administration without assistance. MR-MAP is self-administered by the individual or administered by a caregiver. The vaccination is monitored and supervised by another individual who has received minimal training in observing and recording vaccinations. *Examples: vaccination under teacher or local community leader supervision*.

### 3.5. Use cases validation

As a last step, country **archetypes** were defined to validate the use cases in different settings.

First, the 16 most relevant countries for the measles programme were identified (details provided in Annex 6). Those countries met the following criteria: (i) being among the top 10 most populous countries; (ii) being among the top 10 countries with the highest number of unimmunised children per the latest WUENIC estimates of MCV1 coverage; (iii) being one of the six countries that are identified as a priority for the Measles & Rubella Initiative; and (iv) being one of the six countries that are identified as a priority for Gavi, the Vaccine Alliance. Those countries represent ~70% of the under-5-year-old population using measles or MR vaccine in their routine programmes and can be considered representative of the potential most common use cases for MR-MAPs vaccines.

Second, all the remaining countries were grouped based on their current MCV use and geographic locations. This resulted in four country archetypes (details provided in Annex 7):

- Countries that only use MMR/MMRV. This includes 49 high-income and 32 middle-income countries. These countries have not reported any use of MR or the measles monovalent vaccine since 2012.
- Countries that use MMR/MMRV in their routine schedules report using MR or the monovalent measles vaccine when conducting SIAs, outbreak vaccinations, or additional intensified immunization activities. This includes 33 countries, of which the majority are middle-income countries.
- Countries in the WHO African and Easter Mediterranean regions that only use MR or measles monovalent vaccine. This includes 19 low- and 21 middle-income countries.
- Countries in the WHO's Southeast Asian and Western Pacific regions that only use MR or measles monovalent vaccine. This includes 13 countries, the majority of which are middle-income countries.

Third, use cases were tested for relevance across the 16 priority countries and four archetypes. Based on the analysis of the survey and interview responses, the first four use cases appeared relevant across all 16 priority countries and in the four archetypes. While the self-administration use cases (Use Cases 5 and 6) were often considered not relevant at the current time, the interviews suggested potential in the future across all groups. As a result of this validation step, all six use cases identified were confirmed.

Lastly, they were endorsed by the WHO MR-MAPs Working Group and by the WHO's IVIR-AC.

## 4. Discussion

This first application of a design approach to detailed user research in the fields of vaccinology and vaccination led to the definition and validation of the six use cases for MR-MAP presented above. Putting the users at the center allowed us to comprehensively assess the impact that programmatic aspects and product characteristics will have on the future use of this new product. The output of this process may prove critical for the refinement of the product attributes described in the TPP, the assessment of the potential of MR-MAP, and future vaccine development and implementation decisions.

Although the analysis remains unaffected in terms of its output, it is important to acknowledge two main limitations that affect its scope and reliability:

Given the remote nature of the stakeholder engagement, respondents and interviewees found it difficult to “visualize” the novel MAP features. As a result, their ability to provide strong opinions in some instances was limited. This was particularly true when they were asked to consider the potential use of MR-MAP for a new delivery platform such as self-administration.

It was impossible to directly involve vaccinees and vaccinators (health workers), two key stakeholder groups. Information was collected indirectly via the desk review, survey, and informant interviews; no field study or direct interviews at the service delivery level were conducted. This limited the ability to develop meaningful persona archetypes, allowing the users to validate the use cases, particularly the “new” ones emerging due to innovative MAP product characteristics. It also limited the ability to capture specific needs that may play a role in the design of the product.

Additional vaccine acceptability and implementation research among vaccinees and vaccinators will be required to address these limitations and better understand whether the relevance of the six use cases will change.

Nonetheless, the use cases presented in this article have already served as the foundation for several subsequent activities informing decision-making on MR-MAPs. These included the development of a refined MR-MAP demand forecast (15), where assessing individual use cases across different country archetypes allowed for the definition of more precise assumptions in terms of the target population, MR-MAP market penetration, and coverage growth. The use cases served as the foundation for the development of UNICEF's MR-MAP initial Full Vaccine Value Assessment (iFVVA), with the goal of providing a comprehensive view of the public health and market value of MR-MAPs for public and private stakeholders. As part of the iFVVA work, the use cases provided the basis for disease impact modeling, financial modeling, and, more generally, a discussion about the potential role of MR-MAPs in the MR elimination efforts. Finally, the output of this process confirmed the relevance of the product characteristics described in the WHO-UNICEF's TPP (14), for example, the importance of MR-MAP thermostability and the preference for a MAP that qualifies for use within a controlled temperature chain. Moreover, the need for additional research became apparent to ensure the accurate inclusion of critical programmatic aspects within the TPP. These aspects include exploring the potential for self-administration, addressing policy requirements to enable access and use by CHWs, and finding effective strategies to overcome vaccine hesitancy.

In the future, it is critical to assess how innovations such as MR-MAPs can contribute to achieving the goals of the “Measles and Rubella strategic framework: 2021–2030” and further progress toward eliminating measles. The six use cases provide a user-centric framework for conducting this assessment, taking into account the multiple factors that will influence the future use of this product. The key stakeholders, including users such as caregivers, vaccines, and HWs, remain at the center of this evaluation process.

The initial insights that have emerged during the process of defining the use cases, such as the realization that all use cases could potentially contribute to measles and rubella elimination efforts, have shed some new light on the contribution that MR-MAPs can make and on the potential pathways to make this innovative presentation available. By putting the use cases and the users at the center, we are certain that further research efforts will provide additional insights, which will support not only the development and, hopefully, implementation of MR-MAPs but also enhance the overall effectiveness of the measles programme.

## Data availability statement

The original contributions presented in the study are included in the article/Supplementary material, further inquiries can be directed to the corresponding authors.

## Ethics statement

Ethical review and approval was not required for the study on human participants in accordance with the local legislation and institutional requirements. Written informed consent for participation was not required for this study in accordance with the national legislation and the institutional requirements.

## Author contributions

MH-A, SM, MK, CM, and BG developed the protocol for the study. SM, MK, and CM conducted the interviews and analyses. MH-A managed the project. BG was responsible for securing funding. SM and MH-A wrote the manuscript. All authors reviewed the manuscript, provided technical assistance in its development, have made a substantial, direct, and intellectual contribution to the work and approved it for publication.

## References

[1] Guerra FM Bolotin S Lim G Heffernan J Deeks SL Li Y. The basic reproduction number (R0) of measles: a systematic review. Lancet Infect Dis. (2017) 17:e420–8. 10.1016/S1473-3099(17)30307-9 28757186

[2] World Health Organization. Rubella (German Measles). (2020). Available online at: https://www.who.int/teams/health-product-policy-and-standards/standards-and-specifications/vaccine-standardization/rubella

[3] O'Connor P Jankovic D Zimmerman L Ben Mamou M Reef S. Progress toward rubella elimination - world health organization European region, 2005-2019. MMWR Morb Mortal Wkly Rep. (2021) 70:833–9. 10.15585/mmwr.mm7023a1 34111057 PMC8191869

[4] Motaze NV Mthombothi ZE Adetokunboh O Hazelbag CM Saldarriaga EM Mbuagbaw L. The impact of rubella vaccine introduction on rubella infection and congenital rubella syndrome: a systematic review of mathematical modelling studies. Vaccines. (2021) 9:84. 10.3390/vaccines9020084 33503898 PMC7912610

[5] Causey K Fullman N Sorensen RJD Galles NC Zheng P Aravkin A. Estimating global and regional disruptions to routine childhood vaccine coverage during the COVID-19 pandemic in 2020: a modelling study. Lancet. (2021) 398:522–34. 10.1016/S0140-6736(21)01337-4 34273292 PMC8285122

[6] UNICEF. Measles Cases are Spiking Globally. (2022). Available online at: https://www.unicef.org/stories/measles-cases-spiking-globally

[7] Minta AA Ferrari M Antoni S Portnoy A Sbarra A Lambert B. Progress toward regional measles elimination - worldwide, 2000-2021. MMWR Morb Mortal Wkly Rep. (2022) 71:1489–95. 10.15585/mmwr.mm7147a1 36417303 PMC9707362

[8] World Health Organization. Immunization Agenda 2030 - A Global Strategy to Leave no One Behind. (2020). Available online at: https://www.immunizationagenda2030.org/

[9] World Health Organization. Measles and Rubella Strategic Framework 2021–2030 (2020).

[10] Winter AK Lambert B Klein D Klepac P Papadopoulos T Truelove S. Feasibility of measles and rubella vaccination programmes for disease elimination: a modelling study. Lancet Glob Health. (2022) 10:e1412–e22. 10.1016/S2214-109X(22)00335-7 36113527 PMC9557212

[11] Gavi the Vaccine Alliance. Vaccine Microarray patches (MAPs): Public Summary of the VIPS Alliance Action Plan. (2022). Contract No.: August 29.

[12] World Health Organization. Meeting of the Strategic Advisory Group of Experts on Immunization, October 2016: conclusions and recommendations. Wkly Epidemiol Rec. (2016) 91:561–82.27922031

[13] Hasso-Agopsowicz M Crowcroft N Biellik R Gregory CJ Menozzi-Arnaud M Amorij JP. Accelerating the development of measles and rubella microarray patches to eliminate measles and rubella: recent progress, remaining challenges. Front Public Health. (2022) 10:809675. 10.3389/fpubh.2022.809675 35309224 PMC8924450

[14] World health Organisation and the United Nations Children's Fund (UNICEF). Measles-Rubella Microarray Patch (MR–MAP) Target Product Profile. World Health Organization (2020). Contract No.: ISBN 978-92-4-000020-9.

[15] Ko M Malvoti S Cherian T Mantel C Biellik R Jarrahian C. Estimating the future global dose demand for measles-rubella microarray patches. Front. Public Health. (2022) 10. 10.3389/fpubh.2022.1037157 36726626 PMC9885039

[16] Ivar, Jacobson International,. USE-CASE 2.0 - The Guide to Succeeding with Use Cases 2011. Available online at: https://www.ivarjacobson.com/sites/default/files/field_iji_file/article/use-case_2_0_jan11.pdf

[17] World Health Organization. Immunization and Vaccine- related Implementation Research Advisory Committee (IVIR-AC), Summary and recommendations. (2020). Weekly epidemiological record. 2020(No 49, 2020, 95,), 609–28.

[18] WHO/UNICEF. WUENIC - WHO/UNICEF Estimates of National Immunization Coverage 2022. Available online at: https://immunizationdata.who.int/

[19] Mvundura M Frivold C Janik Osborne A Soni P Robertson J Kumar S. Vaccine innovation prioritisation strategy: findings from three country-stakeholder consultations on vaccine product innovations. Vaccine. (2021) 39:7195–207. 10.1016/j.vaccine.2021.08.024 34412922 PMC8657797

