# Supplementary material for: Exploring potential applications of measles and rubella microarray patches (MR-MAPs): use case identification
Source: Front Public Health. 2023 Jun 12;11:1165110. doi: 10.3389/fpubh.2023.1165110 (PMC10291693; doi:10.3389/fpubh.2023.1165110)
Supplement: Supplementary file 1 [file Data_Sheet_1.docx]

## Identifying use cases for measles and rubella microarray patches (MR-MAPs) - Annexes

#### Annex 1 – additional figures


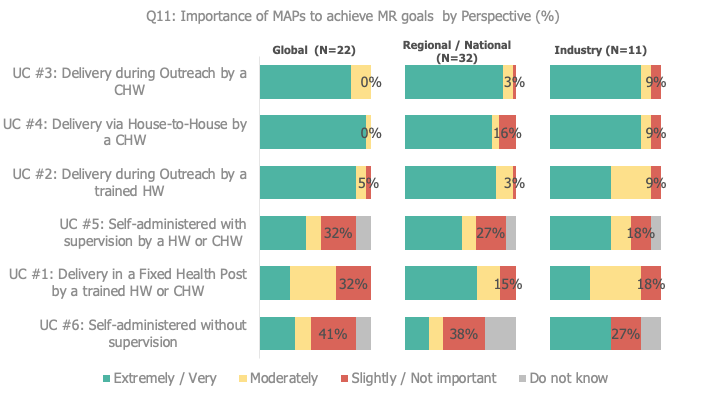


Figure S1: The importance of MAPs to achieve a country’s control and elimination goals by predefined UCs (percentage of respondents)


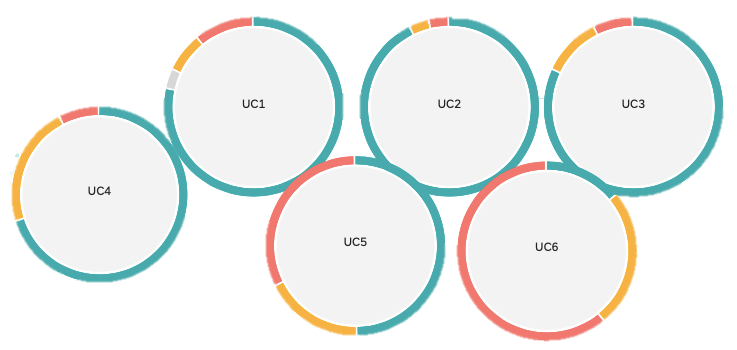


Figure S2: Appropriateness of UCs – output of the interviews

(Notes: green = proportion of positive sentiments, yellow = proportion of neutral, red = proportion of negative sentiments)

#### Annex 2 – Survey methodology and breakdown of participants from the online survey

The project team worked with the WHO IVB team to identify a list of individuals who were engaging in MR activities at the global, regional, and country levels. The survey aimed to obtain general perceptions on the set of defined UCs (Figure 3) as well as obtain feedback to refine their definitions and / or identify additional UCs.

#### A. Survey methodology

A total of 111 individuals were sent personalized links through the Qualtrics™ software and an anonymous link was posted on the TechNet website. Respondents were requested to answer six demographic questions related to their type of organization and their current role in within their organization, current country location, countries referring to when responding to questions, and familiarity with MR control and elimination strategies and MR-MAPs.

Respondents were then requested to answer a series of predefined questions related to a country’s ability to achieving its MR control and elimination goals by: (i) identifying key vaccine delivery challenges and influential factors, (ii) evaluating the importance of the six MR-MAP UCs and where they could be utilised; and (iii) identifying where MR-MAPs would have the most contribution. Respondents were also requested to submit additional programmatic situations where MR-MAPs could be used.

For questions related to sections (i) and (iii) respondents were asked to rate each of the proposed answer using a 5-point Likert scale of “Strongly Disagree” to “Strongly Agree” or “Not [Important / Familiar] at all” to “Extremely [Important / Familiar]”. For certain questions, the option of “Don’t know” was also given. A mean was calculated for each criterion by applying points of 1 for “Strongly Disagree” or “Not [Important / Familiar] at All” to 5 for “Strongly Agree” or “Extremely [Important / Familiar]”. The questions related to section (ii) were matrix questions that evaluated when and where the six UCs would be beneficial. Respondents were provided different types of vaccination programs and World Bank income group classifications and asked to evaluate if MR-MAPs would contribute to the achievement of MR goals by UC. The responses were evaluated by calculating a percentage of the number of individuals indicating MR-MAPs would contribute of the number of total respondents.

The respondents were also given the opportunity to provide additional thoughts for each of the questions. The qualitative responses were individually reviewed and if needed, new categories / topics were created.

An additional stratified analysis was conducted using Microsoft Excel to evaluate any difference in trends in responses stratified by whether the individual was located at the global level, regional/country level, or from industry.

#### B. Survey response rates and demographics

Seventy individuals partially or fully completed the survey. Figure 4 provides the demographics of the respondents by type of organisation, current role, and region. Approximately 47% of the respondents represented an agency of the United Nations followed by 19% who represented industry, product development or design, 13% working in implementation, but not for the UN or a country government, 7% working for a Ministry of Health, and 14% as ‘Other’. Over 35% of the respondents identified themselves as immunization specialists followed by 19% as epidemiologists and ~7-10% each representing the other categories of researchers, EPI managers, surveillance officers, individuals working in development or manufacturing, and ‘Other’. Lastly, survey respondents were asked which country they were based in, with the largest portion indicating their current country within the WHO region of the Americas (31%) followed by the Southeast Asia and African regions at 23% and 21%, respectively, and approximately 7-9% for Eastern Mediterranean, European, and West Pacific regions.

Figure S3: Demographics of survey respondents


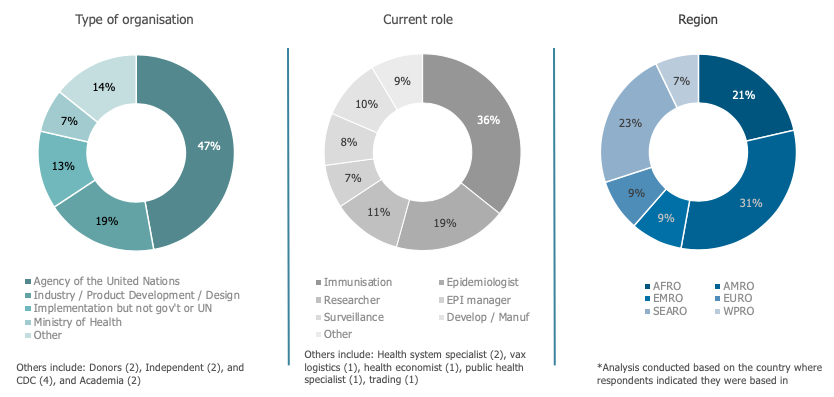


Respondents were also asked their level of familiarity with MR control and elimination strategies and MR-MAPs. Figure 5 provides an overview of the responses with over 70% of respondents indicating to be extremely or very familiar with MR control and elimination strategies while only 17% indicated they were extremely familiar with MR-MAPs followed by 26% and 23% indicating high or moderate familiarity with MR-MAPs, respectively.

The MMGH project team separately reviewed the responses (N=5) for those that selected “Not Familiar at All” to MR-MAPs and deemed that these responses did not significantly affect the overall outcomes or messages. Thus, these responses were maintained in the analysis.

Figure S4: Level of familiarity with MR control and elimination strategies and MR-MAPs


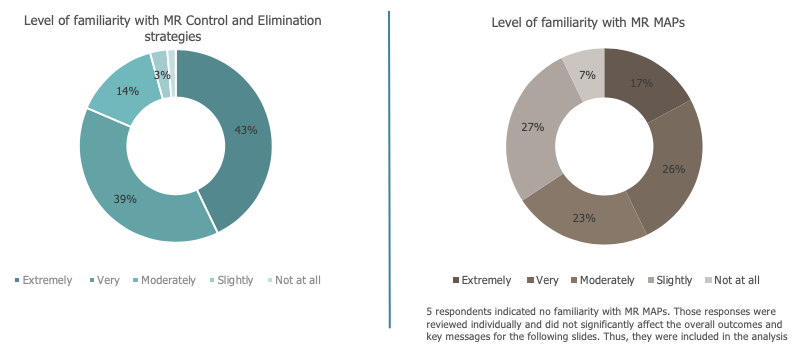


#### Annex 3 - Questions used in the survey

**Q1. What kind of organization do you work for? (Please select the one that applies)**

• Ministry of Health

• Agency of the United Nations

• Implementation agency other than government or United Nations

• Academia

• Industry

• Other (please specify)

**Q2. What role do you have in your organization? (Please select your primary role)**

• Epidemiologist

• EPI manager

• Health systems specialist

• Immunization specialist

• Researcher

• Surveillance specialist

• Other, please describe below

**Q3. What country are you based in?**

▼ Afghanistan (1) ... Other not listed (199)

**Q4. Please select the country(ies) that you are referring to when responding to this survey. To select more than one country, please press the "ctrl" button.**

▼ Afghanistan (1) ... Other not listed (199)

**Q5. How familiar are you with the Measles and Rubella Control and Elimination strategies?**

• Extremely familiar

• Very familiar

• Moderately familiar

• Slightly familiar

• Not familiar at all

**Q6. How familiar are you with the Microarray Patch (MAP) vaccine technology?**

• Extremely familiar

• Very familiar

• Moderately familiar

• Slightly familiar

• Not familiar at all

Microarray Patches (MAPs) consist of hundreds or thousands of tiny projections that deliver dry vaccine into the skin, with some MAPs applied like a bandage and others requiring an applicator for delivery. MAPs have the potential for enhanced heat stability and freeze resistance, increased ease-of-use, for being less painful than an injection and for being sharps-free, thus improving safety.

As such, Measles Rubella MAPs (MR-MAPs) are perceived to significantly ease delivery of MR-containing vaccines, however, they have not yet advanced to clinical development.

The questions below aim at obtaining your input regarding MR-MAPs.

**Q7. In your opinion, do the following delivery challenges impact a country's Measles-Rubella control and elimination goals?**

Available responses: Strongly agree, somewhat agree, neither agree nor disagree, somewhat disagree, strongly disagree.

• Contamination or wastage due to multi-dose vial

• Vaccine ineffectiveness or wastage due to heat exposure

• Reconstitution related safety issues

• Cold chain requirements during outreach

• Needle-stick injuries

• Difficult preparation requiring trained personnel

• Negative impact on the environment due to waste disposal practices

• Reduced acceptability due to painful administration

• Difficult to delivery at correct injection depth

• Other (please specify below)

**Q8. In your opinion, how influential are the following factors on a country's ability to achieve its Measles-Rubella control and elimination goals?**

Available responses: Extremely, Very, Moderately, Slightly, Not influential at all, Do not know.

• Measles endemicity level

• Country income level

• Age of vaccine recipients

• Delivery site (e.g., hospital/dispensary, fixed health post, outreach, mobile, etc)

• Type of immunization provider (e.g., Health worker, Community Health Worker, etc)

• Delivery context (e.g., routine, campaigns, outbreak response, etc)

• Vaccine use (e.g., stand-alone, co-administered)

• Humanitarian emergencies

• Other, please describe below

**Q9. Please describe any other factors that could influence a country's ability to achieve its Measles-Rubella control and elimination goals**

**Q10. In your opinion, how important would MR-MAPs be in achieving the Measles-Rubella control and elimination goals in the country or region in which you work, when considering the following potential uses?**

Available responses: Extremely important, Very important, Moderately important, Slightly important, Not important at all, Do not know.

• Delivery in a Fixed Health Post (e.g., clinic) by a trained Health Worker (HW) or Community Health Worker (CHW)

• Delivery during Outreach by a trained HW

• Delivery during Outreach by a CHW

• Delivery via House-to-House by a CHW

• Self-administered with supervision

• Self-administered without supervision

**Q11. Are there additional programmatic situations in which MR-MAP vaccines could potentially be used? Please describe up to two (2) additional situations, if possible. If you do not know of additional situations, please leave this question blank.**

**Q12. For each situation in which MR-MAPs could be used, please indicate what type of vaccination programme would most benefit from their use.**

Types of vaccination programmes for which responses are sought: Routine Immunization, Periodic Intensification of Routine Immunization, Supplementary Immunization Activities, Outbreak Response Immunization, Delivery outside regular services (e.g., self-administration or pharmacies, etc), Not appropriate to be used in any delivery context, Do not know.

• Delivery in a Fixed Health Post (e.g., clinic) by a trained Health Worker (HW) or Community Health Worker (CHW)

• Delivery during Outreach by a trained HW

• Delivery during Outreach by a CHW

• Delivery via House-to-House by a CHW

• Self-administered with supervision

• Self-administered without supervision

**Q13. Please select all the country context(s) where MR-MAPs could contribute to the achievement of Measles-Rubella control and elimination goals.**

Types of country contexts for which responses are sought: High-income or upper middle-income countries, Non-Gavi lower middle-income countries, Low income & Gavi lower middle-income countries, None, Do not know.

• Delivery in a Fixed Health Post (e.g., clinic) by a trained Health Worker (HW) or Community Health Worker (CHW)

• Delivery during Outreach by a trained HW

• Delivery during Outreach by a CHW

• Delivery via House-to-House by a CHW

• Self-administered with supervision

• Self-administered without supervision

**Q14: Would you agree that MR-MAPs will contribute to the following?**

Available responses: Strongly agree, somewhat agree, neither agree nor disagree, somewhat disagree, strongly disagree, do not know.

• Increase equitable MR vaccine coverage

• Reduce missed opportunities in MR vaccination

• Reduce programmatic errors, and therefore increase safety in MR vaccine administration

• Make transportation of MR vaccines easier

• Reduce MR vaccine cold chain needs

• Reduce MR vaccine wastage

• Reduce Health Worker training needs

• Increase MR vaccine acceptability

• Allow Community Health Workers to administer MR vaccines

• Allow pharmacies to administer MR vaccine

• Allow for self-administration of MR vaccine

• Enhance the convenience of MR vaccine for the recipient

• Increase the reach in insecure / fragile areas

• Ensure timely vaccination response in outbreak situations

**Q15. Please provide any additional comments or feedback.**

#### Annex 4 – Interview methodology and interview responders

The interviews were conducted after the survey (June-July 2020) and aimed to obtain country level perspectives by conducting deep dives into how MR-MAPs could be used in specific country contexts and where MR-MAPs would be most beneficial. The interviews were also used to delve deeper into the rationale of the key trends identified from the survey related to UC1, UC5, UC6, and HICs / UMICs utilizing MR-MAPs.

#### A. Interview methodology

MMGH, in consultation with WHO IVB, selected a set of countries to target for interviews. It was agreed that the countries should be chosen based on the following factors:

- The 10 most populous countries.
- The 10 countries with the most unimmunized children, using WUENIC MCV1 (2019).
- Countries that were previously selected for the Vaccine Innovation Prioritization Strategy (VIPS) deep dive.
- Countries that represent the high-priority countries for Gavi and the Measles & Rubella Initiative.
- A selection of countries that are classified as middle- or high-income countries per World Bank (2019) and
- A selection of countries that are currently experiencing protracted crises.

Based on the criteria above, 49 countries were selected for interviews. MMGH contacted 49 individual EPI managers and interviews were conducted by teleconference using a semi-structured interview guide. Each interview lasted approximately 30 minutes, with the interviewer transcribing the feedback and input. In the situation where MMGH was unable to receive a response from the EPI managers, the WHO immunization focal points were contacted.

The respondents answered a series of predefined questions related to: (i) their current technical MR vaccine delivery challenges; (ii) whether the proposed UCs would be appropriate for the country and why; (iii) additional UCs; (iv) how MR-MAPs would help resolve the previously identified technical vaccine delivery challenges and contribute to the achievement of their MR goals. Questions were sent in advance and, if needed, tailored to the participant. Interviewers documented responses in real time as verbatim as possible.

The senior MMGH team members reviewed all interview transcripts and using an iterative process discussed these to identify the key results and emerging themes. MMGH also utilized TextIQ^TM^ from Qualtrics^TM^, a text analysis tool which assigns topics to qualitative feedback and performs a sentiment analysis (e.g., assigns a positive, neutral, mixed, or negative sentiment to the qualitative feedback). The project team members developed the topics and sub-topics and reviewed all the assigned sentiments, correcting any that were inaccurate. Finally, the project team discussed the results of both analyses to arrive at a consensus and identify the results and emerging themes.

#### B. Interview response rates and demographics

MMGH interviewed 30 individuals across the WHO regions and World Bank Income Group classifications, including 2 WHO regional representatives and officials from 26 countries. The interviewees comprised of 16 EPI managers and 14 WHO immunization focal points at the country or regional levels (MR regional focal points for PAHO and AFRO were interviewed). The figure below provides a map and additional details of the individuals interviewed.

Figure S5: Map of interview respondents


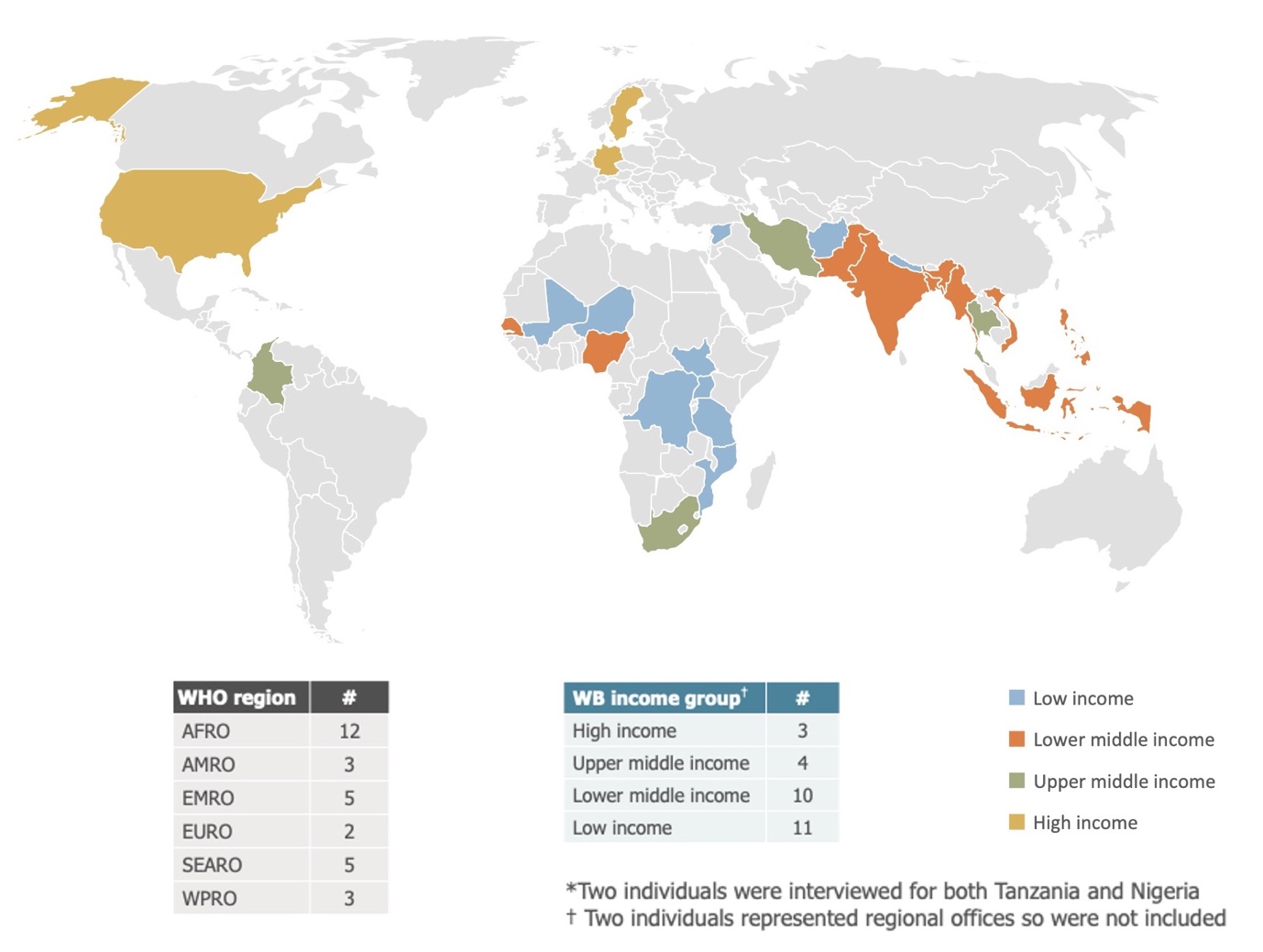


**Annex 5 - Questions used in the interviews**

Q1. What organization do you work for and what is your current role? Which country or country groups will your responses be referring to?

Q2. How familiar are you with the: (i) Regional measles and rubella elimination or control strategies and (ii) the microarray patch (MAP) vaccine technology?

Q3. In your opinion, what are the top 3 technical vaccine delivery challenges that hinder your country’s ability to achieve its Measles-Rubella elimination or control goals?

Q4a. How would your country / country group use MR-MAPs? Where? (e.g., Fixed health post, outreach, other) Who? (e.g., trained individual, untrained individual, self, other) In what context? (e.g., RI, PIRI, SIAs, ORI, outside regular services, other)

Figure 6S: Summary of six MR-MAPs use cases presented to the interviewee


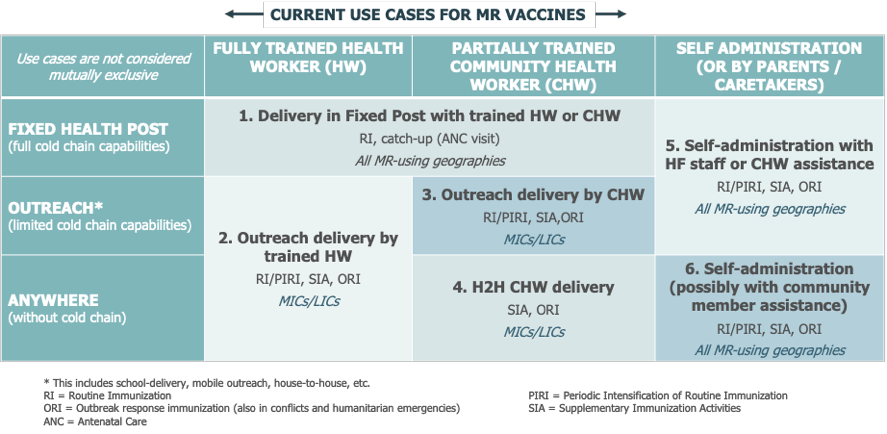


Q4b. In your opinion, which of the above Use Cases for MR-MAPs, are best suited to contribute to achieving your country’s Measles-Rubella elimination or control goals?

Q4c. What is the rationale for your choice?

Q4d. Are there sub-national areas within your country where MR-MAPs would be more useful? If so, please provide specific situations and rationale for why a MAP would be better than the current presentation.

Q4e. For the chosen Use Cases, in what way(s) do you think MR-MAPs can address the vaccine delivery challenges that you previously identified in Q3?

Q5. What would you expect to be the most important programmatic impact of MR-MAPs in your country? And why? 3 categories of potential programmatic impact to be prompted:

- Higher coverage and increased acceptance (e.g., increase equitable MR vaccine coverage, reduce missed opportunities in MR vaccination, enhance convenience of MR vaccine for the recipient, reduce fear of needles)
- Ease of delivery (e.g., reduce programmatic errors, allow community health workers to administer MR vaccines, reach fragile / insecure areas, ensure timely outbreak response)
- Increase effectiveness and efficiencies (e.g., reduce wastage, make transportation of MR vaccines easier, reduce MR vaccine cold chain needs, reduce health worker training needs, allow other potential administration options such as pharmacy or supervised self-administration)

Q6. Do you have any additional comments or any feedback, which you would want to raise at this point?

#### Annex 6 - 16 countries analysed individually

The sizing exercise has a global scope and targets all 194 WHO Member States. 13 small population countries without reported population data from UN WPP were not included

Given the level of uncertainty regarding country use and the need to maintain an efficient yet practical approach, a hybrid model is used to develop and apply the assumptions for 181 countries for each variable. This includes defining a set of most relevant countries that will have unique assumptions; those countries constitute of ~70% of the under 5-year-old population of countries using Measles or MR in their routine programmes and can thus significantly affect the results. Other countries are grouped, and the same assumptions are applied to all countries in that group.

The countries with unique assumptions were selected if they met the following criteria: (i) being among the top 10 most populous countries; (ii) being among the top 10 countries with the highest number of unimmunized children per WHO / UNICEF Estimates of National Immunization Coverage (WUENIC) of MCV1; (iii) being one of the six countries that are identified as a priority for the Measles and Rubella Initiative; and (iv) being one of the six countries that are identified as a priority for Gavi, the Vaccine Alliance.

Figure 7S below provides an overview of the 16 countries that have unique assumptions.

Figure 7S: The selected countries with unique assumptions as part of Step 1


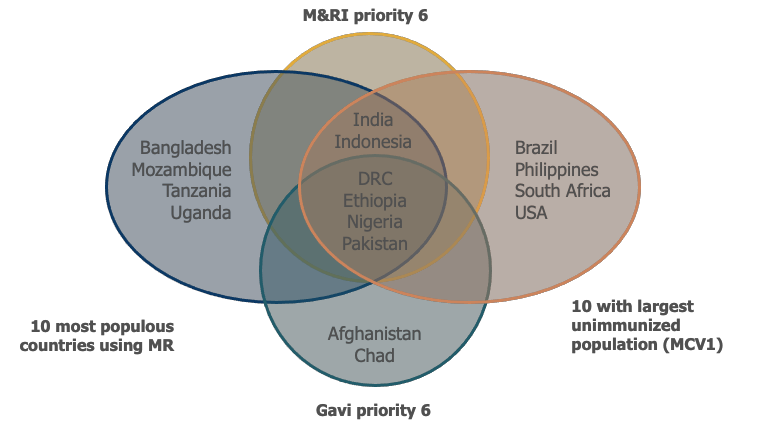


#### Annex 7 - Country archetypes

The 165 countries not analysed individually were grouped based on the country’s use of MCV in 2019 and its geographic locations. The identified country groupings include:

1. **Countries that only use MMR/MMRV**. This includes 49 high-income and 32 middle-income countries. These countries have not reported any use of MR or Measles monovalent vaccine since 2010. (China is currently classified as Group 1 as it has completely moved to MMR and has not conducted any additional immunization activities with MR since 2011).
2. **Countries that use MMR/MMRV in their routine schedule, but report using MR or Measles monovalent vaccine when conducting supplementary immunization activities** (SIA), outbreak vaccination, or additional intensified immunization activities. This includes 33 countries of which the majority are middle-income countries.
3. **Countries in the WHO AFR / EMR regions that only use MR or Measles monovalent vaccine**. This includes 19 low-income and 21 middle-income countries.
4. **Countries in the SEAR / WPR regions that only use MR or Measles monovalent vaccine**. This includes 13 countries, the majority of which are middle-income countries (Japan is currently included in Group 4 as it utilizes MR vaccine in its routine programme).

For the last two groups, countries were grouped by geographic region as a proxy for similar health systems and immunization structures in place.

The list of countries for each of the 4 groups can be found in table 1S

Table 1S: Details on country grouping

| **Country** | **ISO** | **World Bank Group** | **MR MAP group** |
| --- | --- | --- | --- |
| Afghanistan | AFG | Low income | AFG |
| Albania | ALB | Upper middle income | 1 |
| Algeria | DZA | Upper middle income | 2 |
| Angola | AGO | Lower middle income | 3 |
| Argentina | ARG | Upper middle income | 1 |
| Armenia | ARM | Upper middle income | 1 |
| Australia | AUS | High income | 1 |
| Austria | AUT | High income | 1 |
| Azerbaijan | AZE | Upper middle income | 1 |
| Bahamas | BHS | High income | 1 |
| Bahrain | BHR | High income | 1 |
| Bangladesh | BGD | Lower middle income | BGD |
| Barbados | BRB | High income | 1 |
| Belarus | BLR | Upper middle income | 2 |
| Belgium | BEL | High income | 1 |
| Belize | BLZ | Upper middle income | 1 |
| Benin | BEN | Low income | 3 |
| Bhutan | BTN | Lower middle income | 2 |
| Bolivia | BOL | Lower middle income | 2 |
| Bosnia | BIH | Upper middle income | 1 |
| Botswana | BWA | Upper middle income | 2 |
| Brazil | BRA | Upper middle income | BRA |
| Brunei | BRN | High income | 1 |
| Bulgaria | BGR | Upper middle income | 1 |
| Burkina Faso | BFA | Low income | 3 |
| Burundi | BDI | Low income | 3 |
| Cabo Verde | CPV | Lower middle income | 2 |
| Cambodia | KHM | Lower middle income | 4 |
| Cameroon | CMR | Lower middle income | 3 |
| Canada | CAN | High income | 1 |
| CAR | CAF | Low income | 3 |
| Chad | TCD | Low income | TCD |
| Chile | CHL | High income | 2 |
| China | CHN | Upper middle income | 1 |
| Colombia | COL | Upper middle income | 2 |
| Comoros | COM | Lower middle income | 3 |
| Congo | COG | Lower middle income | 3 |
| Costa Rica | CRI | Upper middle income | 2 |
| Cote d'Ivoire | CIV | Lower middle income | 3 |
| Croatia | HRV | High income | 1 |
| Cuba | CUB | Upper middle income | 1 |
| Cyprus | CYP | High income | 1 |
| Czech Rep. | CZE | High income | 1 |
| Denmark | DNK | High income | 1 |
| Djibouti | DJI | Lower middle income | 3 |
| Dominican Rep. | DOM | Upper middle income | 2 |
| DRC | COD | Low income | COD |
| Ecuador | ECU | Upper middle income | 2 |
| Egypt | EGY | Lower middle income | 2 |
| El Salvador | SLV | Lower middle income | 1 |
| Equatorial Guinea | GNQ | Upper middle income | 3 |
| Eritrea | ERI | Low income | 3 |
| Estonia | EST | High income | 1 |
| Eswatini | SWZ | Lower middle income | 3 |
| Ethiopia | ETH | Low income | ETH |
| Fiji | FJI | Upper middle income | 4 |
| Finland | FIN | High income | 1 |
| France | FRA | High income | 1 |
| Gabon | GAB | Upper middle income | 3 |
| Gambia | GMB | Low income | 3 |
| Georgia | GEO | Upper middle income | 1 |
| Germany | DEU | High income | 1 |
| Ghana | GHA | Lower middle income | 3 |
| Greece | GRC | High income | 1 |
| Grenada | GRD | Upper middle income | 1 |
| Guatemala | GTM | Upper middle income | 1 |
| Guinea | GIN | Low income | 3 |
| Guinea-Bissau | GNB | Low income | 3 |
| Guyana | GUY | Upper middle income | 2 |
| Haiti | HTI | Low income | 2 |
| Honduras | HND | Lower middle income | 1 |
| Hungary | HUN | High income | 1 |
| Iceland | ISL | High income | 1 |
| India | IND | Lower middle income | IND |
| Indonesia | IDN | Lower middle income | IDN |
| Iran | IRN | Upper middle income | 2 |
| Iraq | IRQ | Upper middle income | 2 |
| Ireland | IRL | High income | 1 |
| Israel | ISR | High income | 1 |
| Italy | ITA | High income | 1 |
| Jamaica | JAM | Upper middle income | 1 |
| Japan | JPN | High income | 4 |
| Jordan | JOR | Upper middle income | 2 |
| Kazakhstan | KAZ | Upper middle income | 2 |
| Kenya | KEN | Lower middle income | 3 |
| Kiribati | KIR | Lower middle income | 4 |
| Kuwait | KWT | High income | 1 |
| Kyrgyzstan | KGZ | Lower middle income | 1 |
| Laos | LAO | Lower middle income | 4 |
| Latvia | LVA | High income | 1 |
| Lebanon | LBN | Upper middle income | 2 |
| Lesotho | LSO | Lower middle income | 3 |
| Liberia | LBR | Low income | 3 |
| Libya | LBY | Upper middle income | 2 |
| Lithuania | LTU | High income | 1 |
| Luxembourg | LUX | High income | 1 |
| Macedonia | MKD | Upper middle income | 1 |
| Madagascar | MDG | Low income | 3 |
| Malawi | MWI | Low income | 3 |
| Malaysia | MYS | Upper middle income | 2 |
| Maldives | MDV | Upper middle income | 2 |
| Mali | MLI | Low income | 3 |
| Malta | MLT | High income | 1 |
| Mauritania | MRT | Lower middle income | 3 |
| Mauritius | MUS | Upper middle income | 1 |
| Mexico | MEX | Upper middle income | 2 |
| Micronesia | FSM | Lower middle income | 1 |
| Moldova | MDA | Lower middle income | 1 |
| Mongolia | MNG | Lower middle income | 2 |
| Montenegro | MNE | Upper middle income | 1 |
| Morocco | MAR | Lower middle income | 3 |
| Mozambique | MOZ | Low income | MOZ |
| Myanmar | MMR | Lower middle income | 4 |
| Namibia | NAM | Upper middle income | 3 |
| Nepal | NPL | Low income | 4 |
| Netherlands | NLD | High income | 1 |
| New Zealand | NZL | High income | 1 |
| Nicaragua | NIC | Lower middle income | 2 |
| Niger | NER | Low income | 3 |
| Nigeria | NGA | Lower middle income | NGA |
| North Korea | PRK | Low income | 4 |
| Norway | NOR | High income | 1 |
| Oman | OMN | High income | 1 |
| Pakistan | PAK | Lower middle income | PAK |
| Panama | PAN | High income | 1 |
| Papua New Guinea | PNG | Lower middle income | 4 |
| Paraguay | PRY | Upper middle income | 1 |
| Peru | PER | Upper middle income | 2 |
| Philippines | PHL | Lower middle income | PHL |
| Poland | POL | High income | 1 |
| Portugal | PRT | High income | 1 |
| Qatar | QAT | High income | 1 |
| Romania | ROU | Upper middle income | 2 |
| Russia | RUS | Upper middle income | 2 |
| Rwanda | RWA | Low income | 3 |
| Saint Lucia | LCA | Upper middle income | 1 |
| Saint Vincent | VCT | Upper middle income | 1 |
| Samoa | WSM | Upper middle income | 2 |
| Sao Tome | STP | Lower middle income | 3 |
| Saudi Arabia | SAU | High income | 1 |
| Senegal | SEN | Lower middle income | 3 |
| Serbia | SRB | Upper middle income | 1 |
| Sierra Leone | SLE | Low income | 3 |
| Singapore | SGP | High income | 1 |
| Slovakia | SVK | High income | 1 |
| Slovenia | SVN | High income | 1 |
| Solomon Islands | SLB | Lower middle income | 4 |
| Somalia | SOM | Low income | 3 |
| South Africa | ZAF | Upper middle income | ZAF |
| South Korea | KOR | High income | 1 |
| South Sudan | SSD | Low income | 3 |
| Spain | ESP | High income | 1 |
| Sri Lanka | LKA | Upper middle income | 2 |
| Sudan | SDN | Lower middle income | 3 |
| Suriname | SUR | Upper middle income | 1 |
| Sweden | SWE | High income | 1 |
| Switzerland | CHE | High income | 1 |
| Syria | SYR | Low income | 2 |
| Tajikistan | TJK | Low income | 2 |
| Tanzania | TZA | Low income | TZA |
| Thailand | THA | Upper middle income | 1 |
| Timor-Leste | TLS | Lower middle income | 4 |
| Togo | TGO | Low income | 3 |
| Tonga | TON | Upper middle income | 1 |
| Trinidad | TTO | High income | 1 |
| Tunisia | TUN | Lower middle income | 3 |
| Turkey | TUR | Upper middle income | 1 |
| Turkmenistan | TKM | Upper middle income | 1 |
| UAE | ARE | High income | 1 |
| Uganda | UGA | Low income | UGA |
| UK | GBR | High income | 1 |
| Ukraine | UKR | Lower middle income | 1 |
| Uruguay | URY | High income | 1 |
| USA | USA | High income | USA |
| Uzbekistan | UZB | Lower middle income | 2 |
| Vanuatu | VUT | Lower middle income | 4 |
| Venezuela | VEN | Upper middle income | 1 |
| Viet Nam | VNM | Lower middle income | 4 |
| Yemen | YEM | Low income | 3 |
| Zambia | ZMB | Lower middle income | 3 |
| Zimbabwe | ZWE | Lower middle income | 3 |
